# Fostering gender equality and alternatives to violence: perspectives on a gender-transformative community mobilisation programme in rural South Africa

**DOI:** 10.1080/13691058.2019.1650397

**Published:** 2019-08-20

**Authors:** Sarah Treves-Kagan, Suzanne Maman, Nomhle Khoza, Catherine MacPhail, Dean Peacock, Rhian Twine, Kathleen Kahn, Sheri A. Lippman, Audrey Pettifor

**Affiliations:** a Department of Health Behavior, University of North Carolina Gillings School of Global Public Health, Chapel Hill, NC, USA;; b Center for AIDS Prevention Studies (CAPS), Department of Medicine, University of California San Francisco, CA, USA;; c Wits RHI, Faculty of Health Sciences, University of the Witwatersrand, Johannesburg, South Africa;; d School of Health and Society, University of Wollongong, Wollongong, NSW, Australia;; e MRC/Wits Rural Public Health and Health Transitions Research Unit (Agincourt), School of Public Health, Faculty of Health Sciences, University of the Witwatersrand, Johannesburg, South Africa;; f Division of Social and Behavioural Sciences, University of Cape Town School of Public Health, Cape Town, South Africa;; g Sonke Gender Justice, Cape Town, South Africa;; h Department of Epidemiology, University of North Carolina Gillings School of Global Public Health, Chapel Hill, NC, USA

**Keywords:** Violence prevention, South Africa, community mobilisation, gender-transformative programming, Sonke gender justice

## Abstract

Gender-based violence and violence against children are significant problems in South Africa. Community mobilisation and gender-transformative programming are promising approaches to address and reduce violence. A quantitative evaluation of *One Man Can*, a gender-transformative community mobilisation programme in South Africa, found mixed results in increasing gender-equitable behaviours and reducing violence. To better understand these findings, we analyse longitudinal qualitative data from community mobilisers, community members and community action teams, exploring individual and community-level factors that facilitate and hinder change. Interviews and focus groups were transcribed and analysed. Participants self-reported changes in their gender-equitable attitudes and use of violence as a result of participation in the programme, although some participants also reported opposition to shifting to a more gender-equitable culture. Facilitators to change included the internalisation of gender-transformative messaging and supportive social networks, which was buoyed by a shared vocabulary in their community generated by *One Man Can*. Because the programme targeted a critical mass of community members with gender-transformative programming, mobilisers and community action teams were held accountable by community members to model non-violent behaviour. Results reinforce the importance of addressing facilitators and barriers to change at both individual and community levels.

## Introduction

Intimate partner violence, sexual violence and child maltreatment, such as childhood sexual and physical violence, are significant public health concerns in South Africa and are associated with severe health and social consequences (Dunkle et al. 2004; Hatcher et al. 2019; García-Moreno et al. 2013; Richter et al. 2018). Feminist literature attributes gender power inequities, the lower social status of women and surrounding social norms that perpetuate social, economic and political inequality as root causes of violence (Akita 2010; Hunnicutt 2009). Negative gender norms, or the culturally constructed understandings of how men and women are ‘supposed to’ behave, implicitly, if not explicitly in many cultures, condone violence against women and children (Pratto, Sidanius and Levin 2006; Connell 2012). Empirical research consistently finds associations between adherence to negative ideas of masculinity, inequitable gender norms and the perpetration of physical and sexual violence (Fleming et al. 2015). At the individual and interpersonal level, researchers identify violence as a learned behaviour (Bandura 1973); research finds that witnessing domestic violence as a child or experiencing childhood physical or sexual violence is associated with re-victimisation as well as perpetration as an adult (Mulawa et al. 2018; Treves-Kagan et al. 2019). This suggests that violence prevention relies on both challenging norms that condone violence and helping individuals re-learn how to resolve conflict with romantic partners or families without using violence.

Gender-transformative programming is predicated on the assumption that gender norms are malleable and works to help individuals develop more gender-equitable beliefs and behaviours (Gupta 2000). A growing evidence base has found gender-transformative programming to be effective in reducing intimate partner violence perpetration (Dworkin, Treves-Kagan and Lippman 2013). Much gender-transformative programming to date has engaged with men at an individual level or in small group settings. However, qualitative research has found that support, or lack thereof, from peers, partners, families or communities impacts an individual’s adoption of gender-equitable norms (Dworkin, Fleming and Colvin 2015; Gibbs et al. 2015; Gibbs, Jewkes and Sikweyiya 2018). A community-wide approach to encourage a shift towards a culture of gender equality would provide the opportunity for community members to engage with new ideas about gender equality collectively, and bolster individual-level change by simultaneously engaging their networks (Peacock and Barker 2014). Community mobilisation campaigns, such as SASA!,^1^ that use community activities and workshops to raise awareness about the impacts of power inequality and gender norms, have been successful at reducing intimate partner violence (Abramsky et al. 2014).

There is also increasing interest in finding ways to address multiple forms of violence simultaneously. Incorporating anti-violence programming for both men and women – emphasising respect, communication and positive parenting – may serve to reduce intimate partner violence and violence in the home. Given the strong relationship between childhood exposure to maltreatment and domestic violence, and revictimisation and perpetration of violence in adulthood, this synergy merits increased attention.

## *One Man Can* community mobilisation trial

We conducted a cluster randomised control trial to evaluate the impact of *One Man Can*, a gender-transformative community mobilisation programme that challenges gender norms condoning or facilitating violence and sexual risk behaviour and raises consciousness around the intersection of gender norms and HIV (Pettifor et al. 2015; Pettifor et al. 2018; Lippman et al. 2018). The programme was implemented from April 2012 to June 2014 in 11 of 22 villages in the Agincourt Health and Socio-Demographic Surveillance System study area, about 500 kilometres northeast of Johannesburg; the remaining 11 villages were control villages. *One Man Can* was developed and implemented by Sonke Gender Justice (Peacock 2013) and engages men and women in questioning negative norms related to masculinity, gender and power, taking both personal and collective action to build gender equity to end gender violence. The programme also included some content on responsible fatherhood and positive parenting, emphasising alternatives to corporal punishment. The study sought to engage both men and women aged 18–35 years, although adults of any age were welcome to participate in activities.

**Figure 1.**
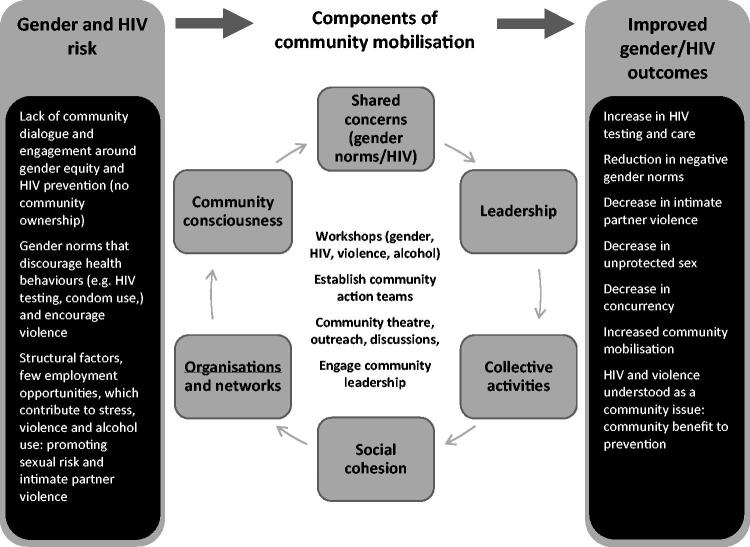
Conceptual framework of intervention, domains of community mobilisation and targeted outcomes (Pettifor et al. 2015).

Community mobilisation aims to change an individual’s behaviour and create a collective that works together to promote change (Evans, Jana and Lambert 2010; Lippman et al. 2013). We defined six domains of community mobilisation: (1) a defined or shared concern that is the focus of change – in this case, gender norms and HIV; (2) raising community consciousness about the issue; (3) an organisational structure with links to groups/networks; (4) leadership (individual and/or institutional); (5) collective or shared activities/actions; and (6) social cohesion, which includes community ties and working trust (Lippman et al. 2013). Figure 1 documents mobilisation domains and their relationship to gender and HIV risk factors and outcomes.

Each participating village had one or two full-time and salaried ‘mobilisers’ and at least one community action team (a group consisting of an average of 5–10 trained volunteers) to implement the intervention activities. Mobilisers and community action teams received extensive training on the intersections of gender, power, HIV and violence prior to commencing work in the community. Over several weeks, mobilisers were trained on intervention content and activities, as well as strategies to teach in an engaging manner and community organising strategies. Intervention leadership and mobilisers met regularly to discuss challenges, document progress towards implementation goals and plan upcoming activities.

**Figure 2.**
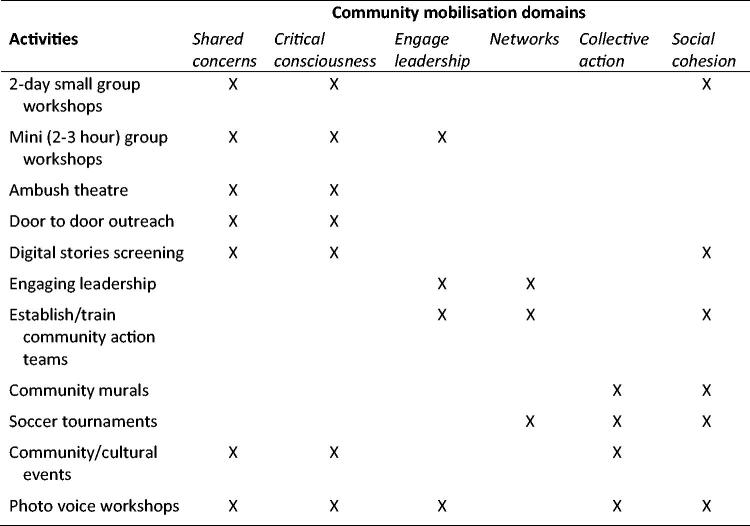
Corresponding intervention activities and community mobilisation domains.

Over the course of the two-year intervention, mobilisers, with the support of the community action teams, led a sequenced series of five different two-day workshops that engaged community members in critically thinking about gender, HIV, violence, alcohol use and sexual behaviour. Community-based activities were also conducted to generate a shared concern and critical consciousness about HIV and gender, including: going door-to-door to discuss a specific theme (safe sex, violence prevention, gender norms etc.); showing digital testimonial stories about violence and HIV to generate dialogue; creating topical murals to create a shared narrative about gender equity and HIV prevention in the community; and conducting group cohesion-generating activities, such as soccer tournaments, where participants joined in discussions focused on gender transformation prior to the event. Mobilisers also engaged local leaders to seek support for *One Man Can* activities and discuss the intervention themes. Figure 2 shows corresponding intervention activities with community mobilisation domains.

As documented in Table 1, over 33% of 18–35-year-old men in intervention communities participated in at least one two-day workshop, and over 5,800 community mobilisation activities were implemented by the end of Year 2 (Pettifor et al. 2018). Some 66% of men and 40% of women had medium to high exposure to the programme, and community social cohesion and critical consciousness increased for people exposed to the programme (Pettifor et al. 2018; Lippman et al. 2018). Between baseline and endline, there were statistically significant improvements in men’s, but not women’s, gender-equitable attitudes. There was some indication of decreased intimate partner violence victimisation among women, but this was not statistically significant (AOR: 0.53, 95% CI: 0.24, 1.16). There was no evidence of reduced male intimate partner violence perpetration in the last 12 months (Pettifor et al. 2018). Quantitative analysis of norms about punishment of children was limited due to limited comparability between surveys.

**Table 1.** Implementation data (May 2012 to April 2014).

	Workshops conducted		Activities conducted		Leader engagement meetings		Community action team meetings		% 18–35-year-old men reached with ≥1 workshop (cumulative)	
	Yr 1	Yr 2	Yr 1	Yr 2	Yr 1	Yr 2	Yr 1	Yr 2	Yr 1	Yr 2
*Larger Villages*										
Community 1	15	27	277	367	8	6	42	44	15%	34%
Community 2	13	26	228	300	8	5	41	44	17%	34%
Community 3	16	23	304	602	12	8	50	77	11%	27%
Community 4	13	22	179	257	2	5	42	43	11%	27%
Community 5	15	29	183	314	7	7	41	49	11%	32%
Community 6	11	27	187	285	7	9	40	46	16%	43%
*Smaller Villages*										
Community 7	8	11	165	314	4	4	36	46	35%	51%
Community 8	11	12	170	236	9	5	41	47	20%	34%
Community 9	12	11	204	328	10	10	42	48	17%	45%
Community 10	9	13	167	294	8	8	41	47	21%	39%
Community 11	12	13	210	259	7	8	43	47	25%	44%
*Total per year*	135	214	2,274	3,556	82	75	459	538	18.1%	37.3%
TOTAL	349		5,830		157		997		37.3%	

## Study aims

**Figure 3.**
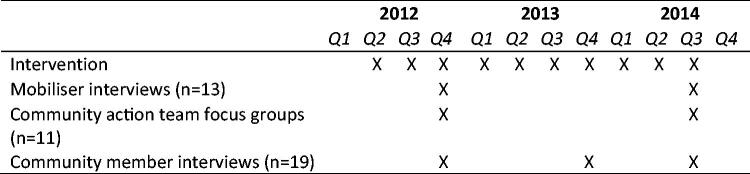
Timeline of intervention activities and data collection.

To complement the quantitative evaluation, the study team collected longitudinal qualitative in-depth interviews and focus groups over the course of 18 months with community mobilisers, community action teams and community members. Data were collected between October 2012 and July 2014; the first round of data collection occurred approximately six months after the start of the intervention, and the last round of data collection occurred as the intervention was ending (see Figure 3). Using this data, we explored participants’ perceived individual and community-level barriers and facilitators to adopting gender-equitable norms and non-violent behaviour. In particular, we examined if, and how, participants’ experiences related to community mobilisation domains.

## Methods

### Sample

Data were collected from 15 community mobilisers, 11 community action teams (124 members total) and 25 community members who had participated in at least one *One Man Can* workshop. Mobilisers participated in two individual in-depth qualitative interviews; community members participated in three individual interviews; and each of the 11 community action teams participated in focus groups at two time points. The sampling strategy was designed to allow for closer examination of the impact of *One Man Can* at multiple levels of engagement. Sample size was determined to allow for thematic saturation.

As described in Table 2, 13 (of 15) community mobilisers completed both interviews (7 males, 6 females). All community action teams participated in both focus groups, although many experienced significant turnover between the rounds of data collection. As such, many participants in the second focus groups had not participated in the initial focus groups. There were a minimum of three and maximum of 10 community action team members represented in the focus group discussions. Nineteen community members completed at least two interviews; 14 completed all three. The main reason for discontinuation in the study was not having time or moving away for employment. Only mobilisers and community members with at least two data points are included in this analysis to be able to examine effects over time.

**Table 2.** Analytic sample description.

Participant Type	Male	Female	Description
Mobilisers (n = 13)	7	6	*One Man Can* employees hired to conduct workshops, community activities, leadership and community engagement
Community action team focus group discussions (n = 11)			
Time 1	24	39	Unpaid volunteer groups supporting the mobiliser’s activities. Focus groups ranged in size from 3 to 9 members. All but two focus groups were mixed gender and generally included more women than men.
Time 2	21	40	
Community Members (n = 19)	11	8	Community members who participated in an initial *One Man Can* workshop were recommended for participation by mobilisers.

Community members ranged in age from 20 to 44 years and included slightly more men (*n* = 11) than women (*n* = 8). Community participants represented four villages almost equally. Community mobilisers were 18–25 years old, equally distributed by gender and represented 10 of the 11 intervention villages.

### Data collection

Local qualitative fieldworkers, all of whom are female, conducted the interviews and focus groups. Fieldworkers were trained in research ethics, study aims, qualitative data collection methods, translation and transcription, and had significant data collection experience in these communities. Semi-structured interview and focus group guides explored participants’ involvement and experience with the intervention, perceptions of personal and community-level change regarding gender norms and violence as well as barriers and facilitators to adopting gender-equitable norms or non-violent behaviour. Interviews and focus groups took 45–90 minutes and were conducted in the local language, Xitsonga. Each fieldworker interviewed the same participants in subsequent rounds to maintain continuity between interviews. Before subsequent interviews, the fieldworker reviewed previous transcripts to allow for reflection and follow-up. Interviews were conducted at participants’ homes or other locations of their choice and were audio recorded. Recordings were translated and transcribed to English by the fieldworker.

### Ethics

Written consent was collected from all participants in their first interview or focus group; verbal re-consent was obtained before subsequent interviews. Confidentiality procedures were discussed at length with participants, in particular with community action teams to ensure members felt safe to share candid thoughts in front of other members, and with community mobilisers, to ensure comfort discussing the *One Man Can* programme and their ability to engage with gender-equitable norms and behaviours. Pseudonyms are used below to protect confidentiality. Ethical approval was provided by the Institutional Review Board of the University of North Carolina at Chapel Hill, the Human Research Ethics Committee of the University of the Witwatersrand, and the Mpumalanga Department of Health and Social Development Research and Ethics Committees.

### Analysis

We used coding, matrices and memos to explore patterns, similarities, differences and trends in our data (Gibbs 2007; Maxwell and Miller 2008). Transcripts were coded in Atlas.ti by two team members. Codes were created a priori, following interview guide questions; some additional emergent codes were added. Coders worked together to establish coding norms and double-coded transcripts until adequate intercoder reliability was established (kappa = 0.73) (MacPhail et al. 2016). Code reports were generated, and a ‘connecting’ matrix was created for each mobiliser, community action team or community member (rows) by time point (columns) with a brief summary of the participants’ report on violence (cells). This facilitated exploring change in attitudes and behaviour over time. Based on this matrix, an initial analysis summary was created for each type of participant (mobiliser, community action team and community member). The first author then reread transcripts, compared data between gender and participant type over time and used memoing to track thoughts on methodological approaches, insights generated while examining the data and how findings related to theory and previous research (Saldaña 2009). Themes identified as fully saturated were explored in the context of the intervention’s community mobilisation framework and the study’s quantitative results.

## Results

Below, and as summarised in Table 3, we describe participants’ perceptions of change in their personal lives and in the community regarding violence. We also reflect on how community mobilisation domains served to help or hinder adoption of non-violent behaviour.

**Table 3.** Qualitative findings as they relate to community mobilisation domains.

Community mobilisation domain	Summary of qualitative results
Collective activities	*One Man Can* led workshops and community activities to engage community members with gender-transformative content with their peers.
Community consciousness	Participants in our sample reported shifts in gender-equitable norms due to *One Man Can*. This was facilitated, in particular, by building respect for women and children and increasing empathy towards victims of violence.
Shared concern	Ambivalence around the acceptability of violence suggest varied success in generating buy-in to gender-transformative messaging that supported alternatives to violence.
Engaging with leadership	Participants reported varied support for gender-equitable norms; lack of support from local structures acted as a barrier to change.
Networks	Participants reported mixed success in engaging their network. When successful, support and accountability were important facilitators to adopting non-violent behaviour.
Social cohesion	A shared vocabulary around gender norms and violence could be a step towards a cohesive community narrative around alternatives to violence, although community-wide buy-in was not fully achieved.

### *Using* One Man Can *activities to raise community consciousness about violence*

*One Man Can* employed gender-transformative workshops and community activities to raise community consciousness around the impacts of negative gender norms, including their relationship to violence. Here a mobiliser discusses how *One Man Can*’s efforts to highlight the issue of violence shifted the community’s perception of the issue:

Violence has been in our community for a long time and people were not discussing it. People were taking it as normal life. So, when we start to discuss it … people start to notice the importance of why we discuss the issue of violence and how violence affects someone’s life. (Rhulani, Male mobiliser, Time 2)

In our data, we found that the most fundamental step in participants’ adoption of alternatives to violence was recognising that women, children and other community members were ‘human beings, like us’ – worthy of respect, consideration and empathy. This then facilitated dialogue and improved communication as an alternative to violence. This sentiment directly maps onto the theoretical underpinning of the *One Man Can* model and social theories understanding of why violence disproportionally impacts some groups over others – with norms that devalue and dehumanise groups of community members serving to justify violence and lack of empathy (Pratto, Sidanius and Levin 2006).

Mobilisers demonstrated significantly more a nuanced understanding and acceptance of these views compared to community members, especially those with less exposure to the programme.

We have made a mistake when we take women as half human; when we are not taking them as human beings like us. (Musa, Male mobiliser, Time 1)

Community members showed substantially more variation in their adoption of gender-equitable norms as compared to project staff and volunteers. Some community members reflected similar sentiments as described earlier:

Now when you look, men understand a lot since *One Man Can* started, they don’t harass us anymore … like my husband now even prepares water for me to bath. Since *One Man Can* started, we have to work equally in everything. Pain is the same and even blood runs in the same veins. So, they understand that they have to treat women well and we also do the same. (Nkateko, Female community member, Time 1)

However, while most participants agreed with gender equality in the context of employment – and that a woman with equal qualifications should be given the same work opportunities as a man – some struggled with ‘disrespecting’ men’s authority in relationships. For example, a male business owner said the following:

[Gender norms] are changing, especially in the households with men who attend *One Man Can* activities. They now understand that a woman is a human being, and she has feelings, and she needs to be involved in decision making *…* (Bongani, Male community member, Time 3)

However, when pressed to clarify how participating in *One Man Can* had, if at all, changed the dynamics with his family, he replied:

Gender equality is a good thing, but there is a place where gender equality needs to be practiced. Like at work, we can apply gender equality … But at home … according to the Bible, gender equality doesn’t fit … because the Bible says, ‘a man is the head of the household’. (Bongani, Male community member, Time 3)

This theme re-emerged when participants discussed barriers to change, with community structures, such as some churches, challenging *One Man Can*’s messaging about what respect and equality should look like within family structures.

### Creating shared concern about violence

Transitioning norms from accepting violence to identifying violence as a shared concern, or a problem that merits community attention and problem solving, is a critical mechanism in using community mobilisation to reduce violence. At all time points, there was general agreement that violence should be avoided. However, some community members showed some ambivalence about the acceptability of violence – for example, providing anecdotes of people being victims of violence even though they didn’t do anything wrong, implying that there may be times when the use of violence might be appropriate. Participants also reported knowing that they were supposed to intervene when witnessing violence by either talking to the victim or perpetrator or involving village leaders, police or social workers. These themes were more evident in second and third interviews.

Individual behaviour change around the practice of violence was reported across participant groups. Mobilisers, some community action team members and some community members reported more easily resolving conflict with their partners, negotiating sex with their partners, adopting positive and non-violent parenting techniques and avoiding other kinds of violence in the community, like bar fights, due to their participation in *One Man Can*. One male community action team member discussed the changes he had made with his siblings and family:

I was unable to keep calm when I was angry. If I argued with my sister, I used to beat her. I used anything that I [could] to throw … to hit the person. At home, I was harsh on children … I sometimes hit the child because I thought the person has disrespected me. Now there is a change because I don’t bother anyone. I do it by myself. I can even cook … (Fumani, Male community action team member, Time 2)

Behaviour change was most evident among the mobilisers, who most frequently discussed arguing less with their partners, not using violence towards their children and siblings and engaging in consensual sex.

For example, eeh … to negotiate sex it was not an easy thing. But now I am able to do it, I am able to negotiate sex and I don’t demand it. (Musa, Male mobiliser, Time 1)

While some community members talked about actions, most were more likely to discuss their intentions to avoid violence or about violence in the community generally. Six community members gave clear examples of reducing violence in their lives, most commonly referencing non-violence towards their children.

I want my children to live with me. I don’t want my children to be scared of me … I want my children to be free when they are with me … When you live with children and shout at them, they will be scared to stay with you … (Nkateko, Female community member, Time 3)

Not wanting her child to be scared of her suggests changing norms about the value of positive parent–child relationships and mirrors participants’ increased empathy towards victims of violence. Community members also discussed reducing violence towards siblings and avoiding fights at local bars as a result of participating in *One Man Can*, which could be interpreted as either an accessible way to engage with *One Man Can* content if one did not have a partner or children or was not previously using violence in those relationships. Alternatively, it might be ‘easier’ to enact non-violent norms with non-partners and children because they operate outside the power dynamics that trigger family violence.

However, participants, especially male mobilisers in their second interviews, acknowledged not always being able to consistently maintain non-violent behaviour. Below, a male mobiliser described a time in the past few months he acted in a way that was ‘counter to *One Man Can*’s ideas about gender equality’:

It is difficult to change, you cannot … it is not an overnight thing … You are not perfect. I’m not either. I argued with my younger brother, and then I promised to beat him, you see. So, I feel like I violated him. And I was angry because I warned him many times to not do what he did, but he didn’t listen to me … So it happened. (Matimba, Male mobiliser, Time 2)

When asked the same question, a female mobiliser reported having challenges maintaining non-violent behaviour towards her child:

Mobiliser: I’m harsh to my child. If he does something wrong, I shout at him. I beat him … and I regret it after I beat him … *One Man Can* is against that. If a child does something wrong, you should sit down with him and talk to him rather than shouting at him.

Interviewer: Ok, so how do you reconcile that with your involvement in *One Man Can*?

Mobiliser: I don’t do it in public, it only happened here in my household and I don’t do it always. It only happened when he did something wrong. (Tintswalo, Female mobiliser, Time 2)

This above quote reflects participants’ struggles to consistently enact non-violent behaviour despite valuing the approach. However, by saying that she only hits her child when he has done something wrong, reflects either ambivalence about never using violence or the need for continued support in adopting new skills.

### Leadership and networks as barriers and facilitators to change

Interviews indicate that engaging *community leadership* and linking to other *groups and networks that provide vehicles for dissemination of messaging and resource sharing*, two additional mobilisation domains, had mixed results. This was most notable with the communities’ local social and leadership structures – like churches, community development forums (local leadership councils) and traditional leaders (i.e. *indunas* – the chief of each village). While there were some examples of working successfully with local leadership, there seemed to be challenges engaging local structures in an ongoing and meaningful way.

While most people reported that the community ‘wanted to live in peace’, meaning living without violence, and was supportive of *One Man Can*’s work in general, all three types of participants reported hearing opposition to some of the tenets of gender equality. Participants reported that cultural norms acted as a community-level barrier to change. Participants repeatedly gave examples of other people criticising men who were operating outside of traditional gender norms (e.g. cooking or taking care of the children), saying that these men had been ‘bewitched’ or ‘charmed’ with witchcraft. Similarly, many reported that religious community members, traditional institutions and leaders or elders called gender-equitable norms ‘white’ or ‘Western’.

There are men who don’t want to swallow the privileges that we were given and face truth. We are trying to engage community members, and there are some who are involved and see that it’s a good thing, but there are still some who thinks it humiliates them. (Musa, Male mobiliser, Time 2)

One female community mobiliser described the challenges she faced in promoting gender-equitable ideas because people felt they were losing an important form of discipline for their wives and children:

They say we are promoting disrespect in the community … You will hear someone saying, ‘you want women to disrespect us – they are no longer punished’. (Tintswalo, Female mobiliser, Time 1)

When specifically asked if there was an organisation that did not support *One Man Can*’s teaching about gender equality, Mandla, a young male community member responded: ‘I can say the culture, tradition. Yes, I can say it is against it’ (Time 2). At the third interview, he repeated this sentiment saying: ‘In the times we live, I will call it culture, eeh … because the culture still believes that dad has to be the head, dad has to lead’.

In addition, as mobilisers’ process of change outpaced that of their peers, this led to isolation. Some male mobilisers also reported not having friends, having friends ‘look at me as a stupid’ or feeling like they no longer belonged in their village but on ‘another planet’. Below a male mobiliser describes the impact of his attitudinal and behaviour change on a previous romantic relationship:

My relationship failed and maybe she didn’t understand me because of the lifestyle I have now. It led me to be single again for 1 year and 6 months now. (Vutivi, Male mobiliser, Time 2)

### A shared vocabulary to bolster a shared concern

While participants reported many barriers to change, they also identified facilitators. A unique aspect of this project was the explicit goal of reaching a critical proportion of community members to facilitate a shift in socio-cultural norms regarding gender equity and parenting practices. Community mobilisation is theorised to enhance collective thought, shared values and movement towards new community standards regarding behaviour and a willingness to support each other through protective behaviours. In the study data, *One Man Can* supported healthy communication skills and seemed to generate a shared vocabulary among study participants, with participants relying heavily on language around respect for women and empathy for victims of violence. These skills and language served to amplify and reinforce new behaviour and renewed linkages in the community.

The relationship between me and my children has changed. Relationships with my friends and my husband have changed. My life when I’m with the whole community has changed. My viewpoints that I share with other people have changed. We learn that everyone is important, and we don’t undermine other people’s views … I don’t say ‘your views are incorrect’ … [instead] I can say, ‘I heard your views, but my views are [different]’. (Ntsako, Female mobiliser, Time 1)

Participants used the language and examples provided by *One Man Can* to talk about, defend or encourage these new ideas and behaviours with their peers, partners, families and community leaders. Community members gave examples of sharing *One Man Can* messages:

After *One Man Can* taught us, we also took this message and spread it further – to tell other people and encourage them. (Solly, Male community member, Time 1)

I always advise my family not to beat children, instead they must talk with them; they must make sure that even the words they are saying to children will not affect them [negatively]. (Kurhula, Female community member, Time 3)

*One Man Can*’s reach within the community, fundamental in creating a community-wide dialogue about violence reduction, was noted in the community action team focus groups and community member interviews. Community members and community action teams perceived that the majority of their community knew about or were ‘touched’ by *One Man Can* programming and were benefiting from it.

There is no one who doesn’t know about *One Man Can*, even old people will tell you about it. (Sipho, Male community member, Time 3)

### Holding mobilisers and community action teams accountable

Unique to their roles in the *One Man Can* intervention, mobilisers and community action teams changed their behaviour in the context of encouraging other people to consider questioning and changing their behaviour. This meant that mobilisers and community action team members went through a difficult and deeply personal process in front of peers, family members and community. This was an explicitly public process as mobilisers and community action teams are, by design, from the communities they were mobilising. Community members often knew mobilisers ‘when their noses were running’ (i.e. when they were children) and how they used to act. They also knew how they were currently acting. This position – of being visible and asking others to change – seemed critical in changing their own behaviour and sustaining that change.

I must lead by example to people that I’m teaching. If I tell things to people, it should be what I’m doing rather than preaching things and not living it. Because when people look at me, they know that I’m living what I tell people. (Ntsako, Female mobiliser, Time 1)

All of the male mobilisers (but almost none of the female mobilisers) identified being held accountable for their actions by the community as a significant motivator for personal change. Being effective in their work relied on ‘walking the talk’ or acting in the way in which they are encouraging other community members to act. Being held accountable for their actions seemed to be a significant driving force in personal behaviour change as mobilisers reported losing community member’s respect if they acted in a way that contradicted *One Man Can* messages.

People will say I’m making them fools because I don’t practice what I preach. (Nhlamulo, Male mobiliser, Time 2)

## Discussion

Quantitative evaluations of the intervention found mixed results in terms of impact on intimate partner violence prevalence. However, participants in this qualitative sample reported meaningful reflection and change regarding gender-equitable norms, non-violent behaviour and changing of social norms more broadly. We found that the most reported change was among interview participants most closely aligned to the intervention, namely the community mobilisers. The mobilisation team had the longest and largest ‘dose’ of the intervention, reflecting potential for deeper internalisation of the *One Man Can* messaging. As has been seen in previous research, our data captured men’s struggle to embrace women’s equality without feeling their own rights infringed upon, especially among community members (Dworkin, Treves-Kagan and Lippman 2013; Dworkin, Fleming and Colvin 2015). This was also reflected in participants’ seemingly easier time reducing other kinds of violence, such as violence against strangers (e.g. bar fights), compared to partner violence or violence against children, as that violence was not connected to maintaining family structures or traditional power hierarchies.

Further, we noted participants’ struggle to sustain non-violent behaviour consistently, despite valuing that approach. In the context of violent behaviour, it may be that workshops and community activities can be important contributors to reducing violence but may not be enough to sustain the change for people with a history of violence. Future work could explore pairing these activities with long-term individual-level programmes that include skills-building. This would be especially critical in intervening in known risk factors for perpetrating violence such as witnessing domestic violence as a child, a history of victimisation, cultural acceptability of violence against intimate partners and alcohol and substance abuse.

Beyond higher exposure to the intervention, mobilisers, particularly men, discussed the importance of being held accountable by those they were teaching to model equitable and non-violent behaviour. These results reflect previous research conducted in South Africa: a study of a school-based HIV peer-education programme found that the programme was not successful because peer educators struggled to maintain their credibility among peers if they (publicly) failed in their own process of transforming inequitable gender norms (Campbell and MacPhail 2002). Our findings provide important implications for future mobilisation and peer-implemented interventions, highlighting the need to train mobilisers extensively through a gender-transformative lens and explicitly address and respond to the complications of delivering an intervention, undergoing personal transformation and living with the programme’s target population.

The scale at which *One Man Can* operated during the project, with participants reporting that the majority of community members had heard about *One Man Can*, was also critical in facilitating a shared vocabulary around gender norms and alternatives to violence. Quantitative data found that 66% of men and 40% of women had medium to high exposure to *One Man Can*, which was less than what participants in the qualitative sample perceived (Pettifor et al. 2018). However, the degree of leaders’ engagement and support of *One Man Can* was mixed, with many participants reporting resistance to adopting gender-equitable behaviour. Similar to previous research, we found that support, or lack thereof, from participants’ broader social networks was critical in facilitating change (Dworkin, Fleming and Colvin 2015; Gibbs et al. 2015; Gibbs, Jewkes and Sikweyiya 2018).

### Limitations and strengths

There are some limitations to this research. There is the risk of social desirability bias; participants may have reported more gender-equitable views or using less violent behaviour because they thought this would be more acceptable to the researchers. This could have been especially true for male participants as all interviewers were female, or for mobilisers or community action teams, given that they were personally vested in the intervention. However, many participants did report their own incidents of violent behaviour or views challenging *One Man Can*’s gender-equitable messaging, suggesting at least some participants were comfortable discussing this topic candidly. In addition, our first round of data collection occurred six months after the intervention started. This limits the ability to see the full arc of change for our participants, particularly as mobilisers had already undergone intensive training. Finally, our sample was purposive to include those who were most engaged over the course of the intervention (i.e. mobilisers and community action team members); we then used a convenience sample of community members that had participated in at least one *One Man Can* event, although further involvement in *One Man Can* activities varied between participants. This sampling approach allowed us to explore change over time but excluded those who were less likely to engage in the intervention and thus less likely to report any behaviour or attitude changes.

Despite these limitations, we believe our findings provide interesting insights to the violence prevention field. For example, quantitatively we saw improvements in men’s gender-equitable norms, and participants in the qualitative sample similarly reported personal changes in their views of gender equality, but highlighted limitations in fully adopting those views. Furthermore, the quantitative evaluation did not find differences in intimate partner violence perpetration in the last 12 months due to *One Man Can*; however, participants in the qualitative sample reported meaningful reductions in violent behaviour. It may be that the frequency or severity of perpetration changed, which would not be captured in a binary variable measuring any intimate partner violence perpetration in the last 12 months.

### Implications for the future

Our findings also serve to inform future research and programming. Results suggest that engaging multiple strategies, focusing on both community and individual-level facilitators to change, will be paramount to programme success. Pairing efforts to cultivate a shared understanding of gender, gender rights and non-violence with skills and support to act on these values is a fundamental building block to violence prevention. Critical to this is engaging with and understanding the cultural norms of a community. Furthermore, this approach can serve to identify strategies that address multiple forms of violence.

## Note

1 SASA! is a community mobilisation intervention, developed by Raising Voices in Uganda, that focuses on shifting norms to reduce intimate partner violence and HIV.

## References

[CIT0001] Abramsky, T., K. Devries, L. Kiss, J. Nakuti, N. Kyegombe, E. Starmann, B. Cundill, L. Francisco, D. Kaye, and T. Musuya. 2014. “Findings from the SASA! Study: A Cluster Randomized Controlled Trial to Assess the Impact of a Community Mobilisation Intervention to Prevent Violence against Women and Reduce HIV Risk in Kampala, Uganda.” *BMC Medicine* 12 (1): 1–17. doi:10.1186/s12916-014-0122-5 PMC4243194 25248996

[CIT0002] Akita, E. 2010. *Hegemony, Patriarchy and Human Rights: The Representation of Ghanaian Women in Politics* (PhD Dissertation). Ohio University, Columbus, OH.

[CIT0003] Bandura, A. 1973. *Aggression: A Social Learning Analysis*. Upper Saddle River: Prentice-Hall.

[CIT0004] Campbell, C., and C. MacPhail. 2002. “Peer Education, Gender and the Development of Critical Consciousness: Participatory HIV Prevention by South African Youth.” *Social Science & Medicine* 55 (2): 331–345. doi:10.1016/S0277-9536(01)00289-1 12144146

[CIT0005] Connell, R. 2012. “Gender, Health and Theory: Conceptualizing the Issue, in Local and World Perspective.” *Social Science & Medicine* 74 (11): 1675–1683. doi:10.1016/j.socscimed.2011.06.006 21764489

[CIT0006] Dunkle, K. L., R. K. Jewkes, H. C. Brown, M. Yoshihama, G. E. Gray, J. A. McIntyre, and S. D. Harlow. 2004. “Prevalence and Patterns of Gender-Based Violence and Revictimization among Women Attending Antenatal Clinics in Soweto, South Africa.” *American Journal of Epidemiology* 160 (3): 230–239. doi:10.1093/aje/kwh194 15257996

[CIT0007] Dworkin, S. L., P. J. Fleming, and C. J. Colvin. 2015. “The Promises and Limitations of Gender-Transformative Health Programming with Men: Critical Reflections from the Field.” *Culture, Health & Sexuality* 17 (supl2): 128–143. doi:10.1080/13691058.2015.1035751 PMC4637253 25953008

[CIT0008] Dworkin, S. L., A. M. Hatcher, C. Colvin, and D. Peacock. 2013. “Impact of a Gender-Transformative HIV and Antiviolence Program on Gender Ideologies and Masculinities in Two Rural, South African Communities.” *Men and Masculinities* 16 (2): 181–202. doi:10.1177/1097184X12469878 PMC3848879 24311940

[CIT0009] Dworkin, S. L., S. Treves-Kagan, and S. A. Lippman. 2013. “Gender-Transformative Interventions to Reduce HIV Risks and Violence with Heterosexually-Active Men: A Review of the Global Evidence.” *AIDS & Behavior* 17 (9): 2845–2863. doi:10.1007/s10461-013-0565-2 23934267

[CIT0010] Evans, C., S. Jana, and H. Lambert. 2010. “What Makes a Structural Intervention? Reducing Vulnerability to HIV in Community Settings, with Particular Reference to Sex Work.” *Global Public Health* 5 (5): 449–461. doi:10.1080/17441690902942472 19507079

[CIT0011] Fleming, P. J., J. McCleary-Sills, M. Morton, R. Levtov, B. Heilman, and G. Barker. 2015. “Risk Factors for Men’s Lifetime Perpetration of Physical Violence against Intimate Partners: Results from the International Men and Gender Equality Survey (IMAGES) in Eight Countries.” *PloS One* 10 (3): e0118639. doi:10.1371/journal.pone.0118639 25734544 PMC4348538

[CIT0012] García-Moreno, C., C. Pallitto, K. Devries, H. Stöckl, C. Watts, and N. Abrahams. 2013. *Global and Regional Estimates of Violence Against Women: Prevalence and Health Effects of Intimate Partner Violence and Non-Partner Sexual Violence*. Geneva: World Health Organization.

[CIT0013] Gibbs, G. 2007. “Comparative Analysis.” Chapter 6. In *Analyzing Qualitative Data*. Thousand Oaks, CA: SAGE.

[CIT0014] Gibbs, A., R. Jewkes, Y. Sikweyiya, and S. Willan. 2015. “Reconstructing Masculinity? a Qualitative Evaluation of the Stepping Stones and Creating Futures Interventions in Urban Informal Settlements in South Africa.” *Culture, Health & Sexuality* 17 (2): 208–222. doi:10.1080/13691058.2014.966150 25335905

[CIT0015] Gibbs, A., R. Jewkes, and Y. Sikweyiya. 2018. “I Tried to Resist and Avoid Bad Friends’ the Role of Social Contexts in Shaping the Transformation of Masculinities in a Gender Transformative and Livelihood Strengthening Intervention in South Africa.” *Men and Masculinities* 21 (4): 501–520.

[CIT0016] Gupta, G. R. 2000. “Gender, Sexuality and HIV/AIDS: The What, the Why and the How.” Paper Presented at *Plenary Address. XIIIth International AIDS Conference*. Durban, South Africa. http://www.med.uottawa.ca/sim/data/assets/documents/DurbanSpeech.pdf

[CIT0017] Hatcher, A. M., A. Gibbs, R. Jewkes, R.S. McBride, D. Peacock, and N. Christofides. 2019. “Effect of Childhood Poverty and Trauma on Adult Depressive Symptoms among Young Men in Peri-Urban South African Settlements.” *Journal of Adolescent Health* 64 (1): 79–85. doi:10.1016/j.jadohealth.2018.07.026 30327276

[CIT0018] Hunnicutt, G. 2009. “Varieties of Patriarchy and Violence against Women: Resurrecting “Patriarchy” as a Theoretical Tool.” *Violence against Women* 15 (5): 553–573. doi:10.1177/1077801208331246 19182049

[CIT0020] Lippman, S. A., S. Maman, C. MacPhail, R. Twine, D. Peacock, K. Kahn, and A. Pettifor. 2013. “Conceptualizing Community Mobilisation for HIV Prevention: Implications for HIV Prevention Programming in the African Context.” *PloS One* 8 (10): e78208. doi:10.1371/journal.pone.0078208 24147121 PMC3795620

[CIT0021] Lippman, S. A., A. M. Leddy, T. B. Neilands, J. Ahern, C. MacPhail, R. G. Wagner, D. Peacock, R. Twine, D. E. Goin, F. X. Gómez-Olivé, et al. 2018. “Village Community Mobilization Is Associated with Reduced HIV Incidence in Young South African Women Participating in the HPTN 068 Study Cohort.” *Journal of the International AIDS Society* 21: e25182. doi:10.1002/jia2.25182 30334377 PMC6192897

[CIT0022] MacPhail, C., N. Khoza, L. Abler, and M. Ranganathan. 2016. “Process Guidelines for Establishing Intercoder Reliability in Qualitative Studies.” *Qualitative Research* 16 (2): 198–212. doi:10.1177/1468794115577012

[CIT0024] Maxwell, J. A., and B. Miller. 2008. “Categorizing and Connecting Strategies in Qualitative Data Analysis.” Chapter 22. In *Handbook of Emergent Methods*. New York: The Guildford Press.

[CIT0025] Mulawa, M., L. J. Kajula, T. J. Yamanis, P. Balvanz, M. N. Kilonzo, and S. Maman. 2018. “Perpetration and Victimization of Intimate Partner Violence among Young Men and Women in Dar Es Salaam, Tanzania.” *Journal of Interpersonal Violence* 33 (16): 2486–2511. doi:10.1177/0886260515625910 26802044 PMC4956596

[CIT0026] Peacock, D. 2013. “South Africa's Sonke Gender Justice Network: Educating Men for Gender Equality.” *Agenda* 27 (1): 128–140. doi:10.1080/10130950.2013.808793

[CIT0027] Peacock, D., and G. Barker. 2014. “Working with Men and Boys to Prevent Gender-Based Violence: Principles, Lessons Learned, and Ways Forward.” Men and Masculinities 17 (5): 578–599. doi:10.1177/1097184X14558240

[CIT0028] Pettifor, A., S. A. Lippman, A. M. Selin, D. Peacock, A. Gottert, S. Maman, D. Rebombo, C. M. Suchindran, R. Twine, K. Lancaster, et al. 2015. “A Cluster Randomised-Controlled Trial of a Community Mobilisation Intervention to Change Gender Norms and Reduce HIV Risk in Rural South Africa: Study Design and Intervention.” *BMC Public Health* 15 (1): 752–759. doi:10.1186/s12889-015-2048-z 26245910 PMC4527273

[CIT0029] Pettifor, A., S. A. Lippman, A. Gottert, C. M. Suchindran, A. Selin, D. Peacock, S. Maman, D. Rebombo, R. Twine, F. X. Gómez-Olivé, et al. 2018. “Community Mobilisation to Modify Harmful Gender Norms and Reduce HIV Risk: Results from a Community Cluster Randomised Trial in South Africa.” *Journal of the International AIDS Society* 21 (7): e25134. doi:10.1002/jia2.25134 29972287 PMC6058206

[CIT0030] Pratto, F., J. Sidanius, and S. Levin. 2006. “Social Dominance Theory and the Dynamics of Intergroup Relations: Taking Stock and Looking Forward.” *European Review of Social Psychology* 17 (1): 271–320. doi:10.1080/10463280601055772

[CIT0031] Richter, L. M., S. Mathews, J. Kagura, and E. Nonterah. 2018. “A Longitudinal Perspective on Violence in the Lives of South African Children from the Birth to Twenty plus Cohort Study in Johannesburg-Soweto.” *South African Medical Journal* 108 (3): 181–186. doi:10.7196/SAMJ.2018.v108i3.12661 30004360

[CIT0032] Saldaña, J. 2009. “Writing Analytic Memos About Narrative and Visual Data.” Chapter 2. In *The Coding Manual for Qualitative Researchers*. Oaks, CA: SAGE.

[CIT0033] Treves-Kagan, S., A. M. El Ayadi, J. L. Morris, L. M. Graham, J. S. Grignon, L. Ntswane, J. M. Gilvydis, S. Barnhart, and S. A. Lippman. 2019. “Sexual and Physical Violence in Childhood Is Associated with Adult Intimate Partner Violence and Non-Partner Sexual Violence in a Representative Sample of Rural South Africans.” *Journal of Interpersonal Violence*: 1–24. doi:10.1177/0886260519827661 30735091

